# Genomic sequencing reveals convergent adaptation during experimental evolution in two budding yeast species

**DOI:** 10.1038/s42003-024-06485-y

**Published:** 2024-07-07

**Authors:** Pu Wang, William W. Driscoll, Michael Travisano

**Affiliations:** 1 Department of Genetics, Cell Biology and Development, University of Minnesota, Minneapolis, MN 55455 USA; 2 Department of Ecology, Evolution, and Behavior, University of Minnesota, Saint Paul, MN 55108 USA; 3 Biology Department, Penn State Harrisburg, Harrisburg, PA 17057 USA; 4 Biotechnology Institute, University of Minnesota, Minneapolis, MN 55108 USA

**Keywords:** Experimental evolution, Population genetics

## Abstract

Convergent evolution is central in the origins of multicellularity. Identifying the basis for convergent multicellular evolution is challenging because of the diverse evolutionary origins and environments involved. Haploid *Kluyveromyces lactis* populations evolve multicellularity during selection for increased settling in liquid media. Strong genomic and phenotypic convergence is observed between *K. lactis* and previously selected *S. cerevisiae* populations under similar selection, despite their >100-million-year divergence. We find *K. lactis* multicellularity is conferred by mutations in genes *ACE2* or *AIM44*, with *ACE2* being predominant. They are a subset of the six genes involved in the *S. cerevisiae* multicellularity. Both *ACE2* and *AIM44* regulate cell division, indicating that the genetic convergence is likely due to conserved cellular replication mechanisms. Complex population dynamics involving multiple *ACE2/AIM44* genotypes are found in most *K. lactis* lineages. The results show common ancestry and natural selection shape convergence while chance and contingency determine the degree of divergence.

## Abstract (web summary)

In this study, the authors retrieve frozen stocks of the *K. lactis* lineages and reconstruct the evolutionary dynamics of parallel lineages at the genetic level with high throughput sequencing

Convergent evolution is widely observed, such as in streamlining in aquatic animals^1,2^. Leveraging observations of convergence is a powerful approach to investigate evolutionary processes underlying adaptation and divergence, particularly among repeated examples of adaptive radiation in Darwin’s finches^3,4^, *Anolis* lizards^5^, and sticklebacks^6^. These and other studies have demonstrated the potential for natural selection to lead to predictable evolutionary outcomes, at least in principle^7,8^. Among distantly related species, convergence is frequently attributed to adaptive responses to shared environments^9^. At the same time, convergence is also associated with shared genetic background and the underlying mechanisms involved in adaptation, because evolutionary outcomes have a strong phylogenetic component^1,2,10^. Determining the genetic basis for convergence is central to disentangling the contributions of shared environment and shared ancestry.

In the history of life, one of the most important occurrences of convergence is the evolutionary origin of multicellularity^11^. Multicellularity independently evolved over 25 times across the tree of life^11,12^. Its repeated evolution, from diverse ancestors and in different environments, strongly suggests that it conveys substantial selective benefits^13^. However, in this case, the extraordinary diversity of origins and environmental circumstances in the convergent evolution of multicellularity severely limits our ability to disentangle its evolutionary causes. Insights from investigating the genetic mechanisms are challenging since confounding influences are likely and it is difficult to disentangle their contributions^12,14^.

The experimental evolution of two snowflake yeast species provides a comparative experimental approach for investigating convergent multicellular evolution^15,16^. Applying settling selection, which favors larger particle sizes that settle faster in liquid, on the diploid unicellular yeast *Saccharomyces cerevisiae* led to the evolution of a multicellular form, the snowflake phenotype, revealing a mechanism of multicellular evolution^16^. A similar selection of another yeast, the haploid *Kluyveromyces lactis*, also led to the evolution of snowflake phenotypes (Fig. 1) ^15^. While both organisms are budding yeasts with similar single cell phenotypes, *S. cerevisiae* and *K. lactis* differ phylogenetically by more than 100 million years of divergence, including a genome duplication in the lineage leading to the *S. cerevisiae*^17,18^.

**Fig. 1:**
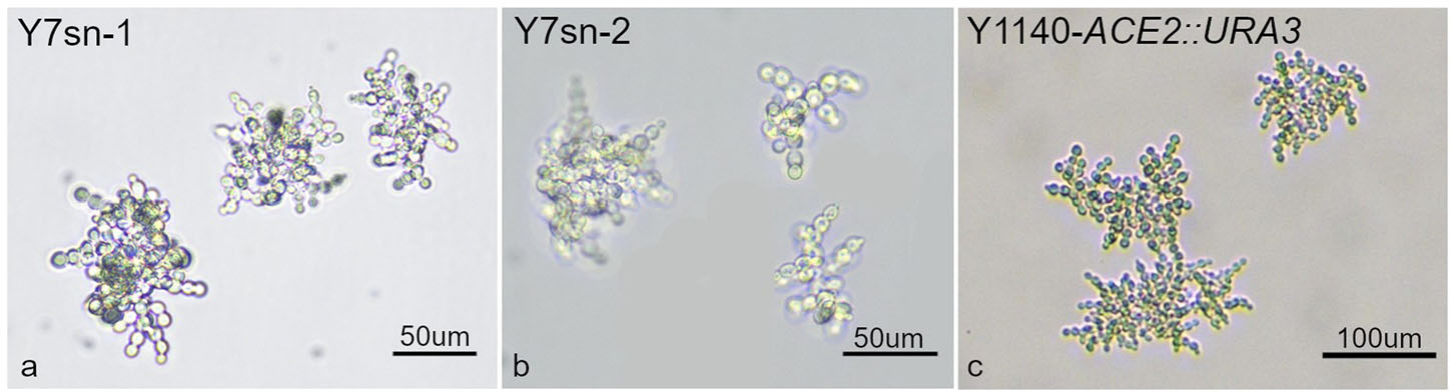
Snowflake structures of representative strains. **a**, **b** Y7sn-1 was isolated from the evolving Y7 population on Day 60 and Y7sn-2 was isolated from the Day 10 glycerol stock. Y7sn-2 clusters are more branchy and of smaller sizes compared to Y7sn-1. **c** The *ACE2* gene was knocked out from the Y1140 ancestral genotype, which resulted in a snowflake phenotype.

We have the complete archive of these experimentally evolved yeasts, which can be easily revived to review the evolutionary history at multiple time points. In this study, we retrieve frozen stocks of the *K. lactis* lineages obtained by Driscoll and Travisano^15^ and reconstruct the evolutionary dynamics of parallel lineages at the genetic level with high throughput sequencing. We then compare the genetic mechanisms of *K. lactis* multicellularity to that of previously investigated *S. cerevisia*e^16,19^. Evolutionary trajectories are reviewed using whole genome sequencing of 75 revived “fossils”, including 60 preserved, historical *K. lactis* populations for ten parallel lineages (samples preserved every 10 days for 60 days), 14 single genotype isolates (13 terminal), and the ancestor.

We observed strong genetic convergence between *K. lactis* and *S. cerevisiae* in the evolution of snowflake multicellularity, regardless of their numerous phenotypic, genomic and evolutionary differences. Loss of function mutations in either of two loci, *ACE2* and *AIM44*, conferred multicellular phenotypes in *K. lactis*, and both loci were similarly identified during the evolution of *S. cerevisiae* snowflake multicellularity. The gene products of both loci are regulators involved in cell division, suggesting that the observed genetic convergence is due to conserved mechanisms of cell replication in budding yeast in the shared selective environment. However, the phenotypic and genetic convergence between *K. lactis* and *S. cerevisiae* belies complex multiple levels of divergence and convergence. As previously reported, we observed coexistence of unicellular and multicellular individuals *K. lactis* populations^15^ while snowflakes completely outcompeted unicells in *S. cerevisiae* populations^16^. This divergent evolutionary response of unicellular and multicellular coexistence in *K. lactis* populations provided opportunities for additional multicellular lineages to evolve from coexisting unicells in the majority of *K. lactis* populations. Interactions among multiple *ACE2* mutants were observed in seven out of ten populations. Our work demonstrates convergent genetic evolution is strongly influenced by conserved mechanisms and common ancestry, but its limitation of predictability is due to chance and contingency arising over the course of evolution.

## Results

We focused on the haploid *K. lactis* multicellular populations selected by Driscoll and Travisano^13^. In all ten replicate *K. lactis* populations, unicellular and multicellular phenotypes were able to co-exist after settling selection, due to frequency-dependent selection^15^. To determine the genetic basis of the evolved *K. lactis* multicellular forms, we first obtained single terminal multicellular isolates from each of the Day 60 populations, three additional terminal isolates from populations Y1, Y8 and Y9, and the initially appearing snowflake genotype (from Day 10) from population Y7. We sequenced the 14 single genotypes as well as the ancestral strain. Furthermore, population genomic sequencing was conducted on every preserved historical population from Day 10 to Day 60 to reveal the evolutionary dynamics of each population at the genetic level. Altogether, we have acquired genomic information of 14 multicellular genotypes, 60 historical populations, and the ancestral strain. Genomic sequencing results revealed highly convergent evolution at the genetic level, consistent with the observed phenotypic multicellular convergence in *K. lactis* (Table. 1).

**Table 1:** Description of mutations within snowflake phenotypes in ten populations

Population	Strain	Phenotype	Gene	Ref	Alt	Annotation
1	**Y1sn-1**	**snowflake**	*ACE2*	C	A	E466*
1	**Y1sn-2**	**snowflake**	*ACE2*	G	A	Q49*
2	**Y2sn-1**	**snowflake**	*ACE2*	G	A	Q480*
3	**Y3sn-1**	**snowflake**	*ACE2*	G	A	Q480*
3	Y3sn-2	putative snowflake	*ACE2*	G	T	H 713 N
4	**Y4sn-1**	**snowflake**	*ACE2*	C	A	E236*
4	Y4sn-2	putative snowflake	*ACE2*	G	T	H 713 N
4	Y4sn-3	snowflake	*ACE2*	T	TC	Q49fs, 56*
5	**Y5sn-1**	**snowflake**	*ACE2*	T	TC	Q49fs, 56*
5	Y5sn-2	snowflake	*ACE2*	G	T	C605*
6	**Y6sn-1**	**snowflake**	*ACE2*	T	TC	Q49fs, 56*
7	**Y7sn-1**	**snowflake**	*ACE2*	T	TC	Q49fs, 56*
7	**Y7sn-2**	**snowflake**	*ACE2*	T	C	M1?, start codon
8	**Y8sn-1**	**snowflake**	*ACE2*	A	AT	N231K, 232*
8	**Y8sn-2**	**snowflake**	*ACE2*	T	C	M1?, start codon
9	**Y9sn-1**	**snowflake**	*ACE2*	G	A	Q371*
9	Y9sn-2	snowflake	*ACE2*	TC	T	D833fs, 845*
9	Y9sn-3	snowflake	*ACE2*	G	A	Q504*
9	**Y9sn-4**	**snowflake**	*AIM44*	CAT	C	S113fs, 131*
10	**Y10sn-1**	**snowflake**	*ACE2*	G	A	Q190*? Not detected in the pop data
10	Y10sn-2	snowflake	*AIM44*	CAT	C	S113fs, 131*
10	Y10sn-3	putative snowflake	*ACE2*	G	T	synonymous
10	Y10sn-4	snowflake	*ACE2*	G	A	R622*
10	Y10sn-5	snowflake	*ACE2*	TC	T	G48fs, 67*

Phenotypes of bolded strains were confirmed by isolating and microscopic observation, with isogenic genomes sequenced.

*indicates stop codon, ? indicates unusable start codon.

### The genetic basis of multicellular forms

Mutations in the *ACE2* gene were found in 12 of 13 terminal *K. lactis* snowflake multicellular isolates (Fig. 1), including one from each replicate population, after 60 days of selection strains (Table 1). The *K. lactis ACE2* protein is 854 aa in length, with a zinc finger region between 686 and 761. The majority of mutations were stop codons or frameshifts resulting in the truncation of *ACE2* and the loss of a zinc finger. In one isolate, we observed a shift to a much less efficient start codon (AUG to GUG). All variants are anticipated to be complete or partial loss of function alleles and a mutant strain constructed by knocking out *ACE2* in the ancestral background has a similar snowflake phenotype (Fig. 1c). One multicellular strain, Y9sn-4, had a 2 bp deletion in *AIM44*, which is anticipated to be the cause for the phenotype since loss of functions mutations in *AIM44* were previously reported in *S. cerevisiae* snowflakes^20^.

Aside from the *ACE2* and the *AIM44* mutations, three other detectable mutations were observed. Y1sn-1 had a novel synonymous mutation in *KLLA0F12276g* (G3288A, unknown protein function). This variant was not observed in the population sequencing. Different frameshift mutations in the *CIP1* locus were observed in Y8sn-2 and Y9sn-4, in addition to their respective *ACE2* and *AIM44* mutations. Both of these *CIP1* variants were detected in the population sequencing well after the multicellularity arose in each population (Days 50 and 40, respectively). Thus, the snowflake phenotypes in all our populations resulted from mutations in either *ACE2* or *AIM44*.

### The evolutionary dynamics of multicellular forms in *K. lactis*

The high degree of genetic convergence across replicates allowed us to use the *ACE2* and *AIM44* genes as markers to trace the evolutionary history of multicellular across the populations. The evolutionary changes that occurred over the 60-day period could be captured by examining the frequencies of *ACE2* and *AIM44* mutants in the historical frozen populations which were preserved at 10-day intervals during selection. Evolutionary trajectories were reconstructed using genomic sequencing data of 60 preserved populations and the ancestor. This also allowed for a thorough and rigorous screen for all mutations.

**Fig. 2:**
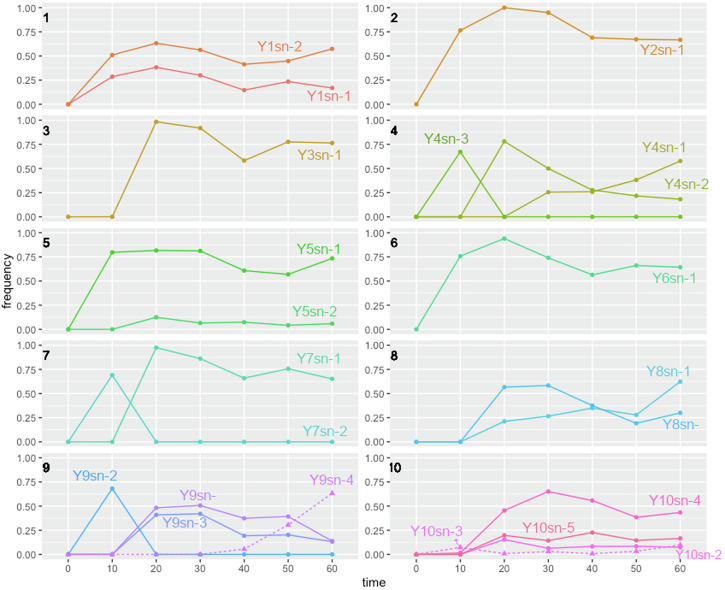
Ten parallel evolving populations of *K. lactis* were studied to explore different patterns of multicellular evolution dynamics. For example, in population Y1, two multicellular genotypes coexisted while only a single snowflake genotype was observed in Y2. In Y7, an early-evolving genotype was subsequently replaced by a later one. Solid line: *ACE2* mutants, dashed line: *AIM44* mutants. Refer to the Supplementary Table 1 for the values of genotype frequencies.

**Fig. 3:**
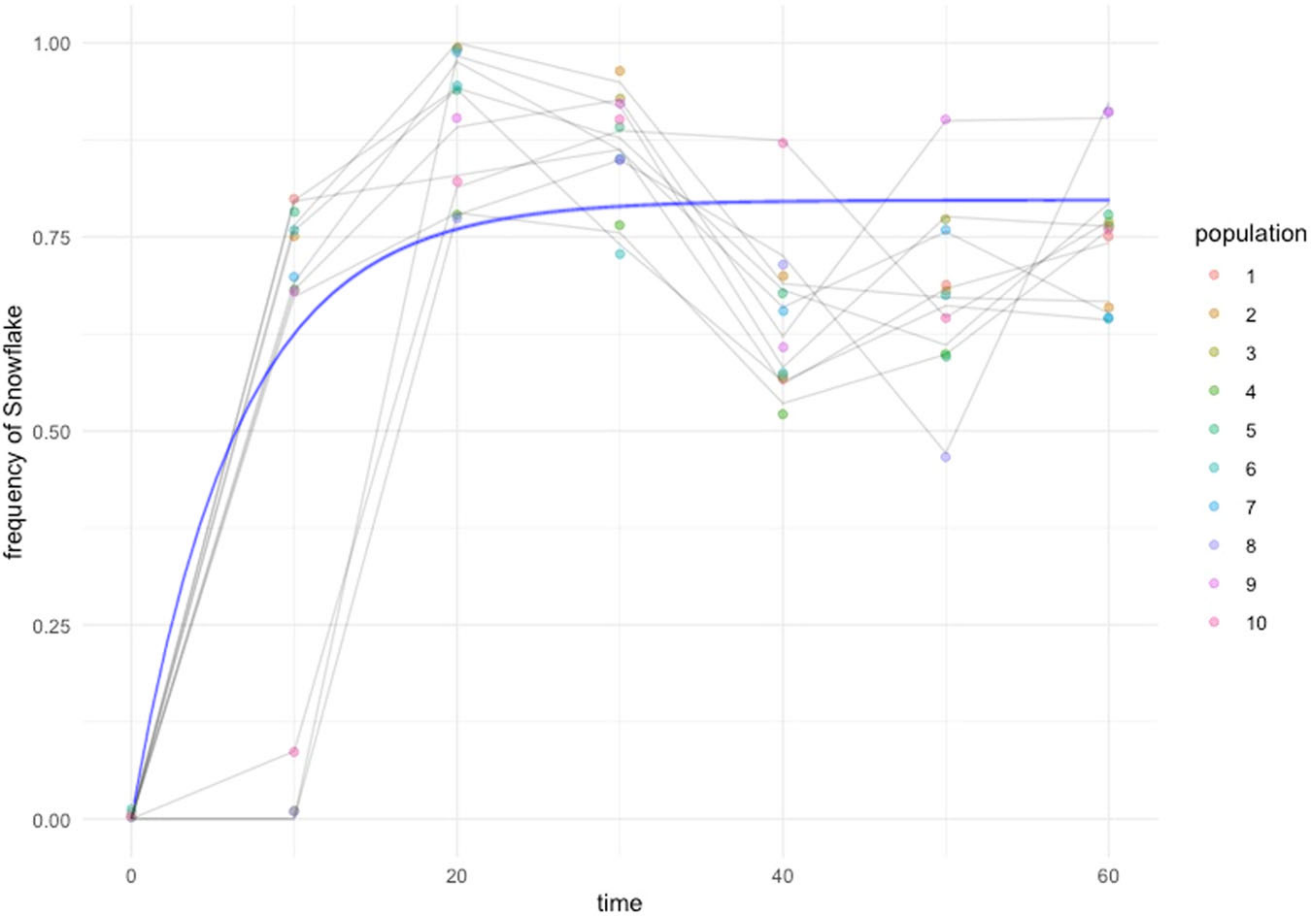
The trajectory of the total frequency of snowflake alleles through time. New *ACE2* and *AIM44* mutant genotypes rapidly emerged within 10 days leading to the increase of snowflake frequencies. However, as more new genotypes emerged rapidly in the next 10 days, snowflake frequencies remained stable frequencies, indicating the competition among snowflake genotypes and frequency-dependent selection. Modeling of multicellular genotype evolutionary dynamics over 60 days of settling selection. The predicted model: frequency=0.766-0.771*exp(−0.194*time). *R*^2^ = 0.729.

*ACE2* and *AIM44* variants appeared and arose rapidly in each population, totaling an average of 44.5% in each population after roughly 10 days of propagation (Figs. 2, 3). Additional new snowflake mutants appeared after the initial ten days but contributed little to the increase of multicellular allele frequencies across populations (Fig. 3). The majority of the variant alleles appeared within the first 20 days of selection, and only two more arose afterward (Fig. 3). Similar to our findings for the multicellular isolates, the vast majority of *ACE2* mutations observed are expected to be loss of function due to stop codons, except for one nonsynonymous mutation (H713N) in population Y4 and one synonymous mutation (among the four variants) in populations Y10. Certain positions within the gene had a higher observed number of mutations due to the nucleotide content. For example, six populations had mutations at 143 bp from the start codon of *ACE2* which we identified as a poly-C (Cx5) simple-sequence repeat. Mutations leading to the loss of function in *AIM44* were detected in Y9 and Y10 populations with both involving the same frameshift mutation.

As shown in Figs. 2 and 3, the total snowflake allele frequency of *ACE2* and *AIM44* mutants rapidly stabilized across the *K. lactis* populations during the selection and no further consistent change was observed following the initial 10 days of propagation (REML ANCOVA, adj *R*^2^ = 0.830, *F*_1,9_ = 0.261, *p* = 0.622). While the evolutionary trajectories of all ten populations show a high degree of convergence for snowflake conferring alleles, especially *ACE2*, diversity and divergence was also observed. Statistically significant variation in allele frequencies across populations was detected following the initial 10 days of propagation (Wald *p* = 0.0483), indicating that populations differed in the tempo of total snowflake allele frequencies. Multiple multicellular conferring alleles were observed over the course of selection in eight of the ten populations (Table 1), with only one snowflake allele observed in populations Y2 and Y6 (Fig. 2). In two populations, the newly evolved genotype replaced the earlier genotype (Y3 and Y7, Fig. 2). In contrast, multiple genotypes co-existed in four populations (Y1, Y5, Y8 and Y10, Fig. 2). In the remaining two populations, both replacement and coexistence were observed (Y4 and Y9, Fig. 2). We did not observe any apparent relationship between the position of mutations within *ACE2* and the persistence of the associated snowflake genotype. Even though similar mutations were detected in several populations, the evolutionary outcome differed, with some genotypes persisting and others being replaced (e.g., Y7sn-2 and Y8sn-2, with the same *ACE2* mutation).

### Coexistence and exclusion due to cluster size

The phenotypic features of evolving snowflakes were also examined following revival from historical frozen stocks. In most populations, snowflakes could be observed in stocks preserved as early as Day 10, the earliest time when frozen stocks were created. This reflects the rapid emergence of multicellular phenotypes in response to settling selection. Most populations had more than one snowflake allele appearing over the course of selection, with the early-appearing alleles either being replaced or coexisting with later ones. To investigate the basis for replacement or coexistence, we chose to study the settling ability of variants in two populations, Y1 and Y7. In Y1, two *ACE2* variants, Y1sn-1 and Y1sn-2, were initially observed in the Day 10 population sample, and they co-coexisted throughout the selection to Day 60. In Y7, the Y7sn-2 variant emerged within the first 10 days but was quickly replaced by the Y7sn-1 variant that persisted throughout the rest of the selection experiment.

For each population, we conducted a co-settling tests of both variants. The two snowflakes from each population were mixed together to determine relative settling rates. Strains were distinguishable from each other through the insertion of an uracil marker. Since the data were not normally distributed, Wilcoxon signed-rank tests was conducted. In population Y1, in which both variants coexisted, no statistically significant differences in settling were observed between Y1sn-1-*URA3*Δ strain and Y1sn-2 (SRC = −0.107, df = 4, *p* = 0.813). In contrast for population Y7, Y7sn-1-*URA3*Δ settled significantly better than Y7sn-2 (SRC (Y7sn-2 - Y7sn-1) = −0.424, df=5, *p* = 0.031), consistent with the observed evolutionary trends in the selection experiment.

We then measured the particle sizes of each strain using a Coulter Counter cell sizer, and assessed if the competitive outcome of snowflakes, exclusion or co-existence, was likely driven by differences in snowflake cluster size. We observed statistically significant differences in size for both populations, and the differences were significantly greater in population Y7 in comparison with Y1. In Y7, there was a 4.3um ± 0.63um (95% CI) size difference between isolates while there was only 1.2um ± 0.63um (95% CI) difference in Y1. This is consistent with individual snowflake size having a selective impact, but it not being the sole determinant of fitness. Indeed, the differences among populations Y1 and Y7 for isolate size, 2.9um ± 0.45um (95% CI), is greater than that between the isolates in Y1 suggesting that multicellular cluster size is only one factor affecting evolutionary outcomes. In Y7, the Day 10 Y7sn-2 clusters were smaller, less circular in shape, and had a branching appearance (Y7sn-2 diameter 20.39 μm ± 6.33 μm, Supplementary Table 3), whereas the Day 60 clusters formed a bigger and less branchy snowflake (Y7sn-1 diameter 24.51 μm ± 4.53 μm, Supplementary Table 3). Snowflake settling rates are positively correlated with size and snowflake shape^16,19^, morphological differences are likely the basis for faster settling and exclusion by the later arising snowflake genotypes.

### No evidence for changes in floccing among genotypes

Changes in floccing, adhesion of snowflakes and unicells to one another, can potentially alter competitive ability through changes in settling. Floccing of multicellular snowflakes is a substantial determinant of settling ability in *K. lactis* and floccing of unicells to snowflakes is the basis for unicell coexistence^15^. Floccing ability within and among genotypes was investigated by microscopic image analysis. Each imaged population was composed of 50% stained and 50% unstained snowflakes either from the same genotype (e.g. Y7sn-1) or two different genotypes (e.g. Y7sn-1 and Y7sn-2). If snowflakes had a higher tendency to floc within one genotype, there would be significantly more one-colored clusters in populations composed of two genotypes while populations with just one genotype would show no significant differences among the cluster color patterns. A Chi-square test was used for each population measurement and showed no significant differences in floccing patterns in two-genotype populations (Supplementary Table 4, *p* values > 0.05 after Bonferroni correction for multiple simultaneous tests). This suggests that evolved snowflakes floc indiscriminately.

## Discussion

We observed strong phenotypic and genetic convergence between *K. lactis* and *S. cerevisiae* populations in the evolution of snowflake multicellularity. The snowflake phenotype confers fitness benefits during settling selection, and adaptive evolutionary responses largely influenced the observed convergent evolution. Reconstructing the evolutionary history of the replicate *K. lactis* populations using population genomic sequencing showed that *K. lactis* multicellularity was caused by mutations in either of two genes *ACE2* and *AIM44*, with *ACE2* being predominant. These two genes are a subset of the six genes involved in the origin *S. cerevisiae* snowflake multicellularity^20^. There was substantially more parallelism and convergence for snowflake multicellularity in the *K. lactis* populations, than *S. cerevisiae*, as *ACE2* variants were identified in every *K. lactis* lineage. Hence, there is divergence in the degree of parallelism within species over the course of selection. The dynamics and appearance of *ACE2* variants in the *K. lactis* populations suggests that the persistence of unicellular phenotypes and the ploidy of *K. lactis* were also important in the degree of convergence and parallelism.

### Conserved mechanisms facilitate convergence

Natural selection acts directly on phenotypes. Genetic convergence in response to shared environmental conditions is indicative of shared physiological and genetic mechanisms of adaptation^8,21,22^. Both yeast species responded similarly phenotypically by forming larger clusters via the failed separation of daughter cells. This is likely due to budding mechanisms of cellular reproduction common to both yeasts. Thus, the shared ancestry of reproduction was a strong determinant of the similar phenotypic evolutionary responses to the same selective force^8^. This convergence also occurred genetically. Gettle^20^ found that mutations leading to the loss of function in the six target genes, including *ACE2* and *AIM44*, led to the same ultimate mechanism, down regulation of chitinase and glucanase, resulting in the failure of separation of daughter to mother cells during budding^23,24^. While the *K. lactis* genome is less well annotated than *S. cerevisiae*, *ACE2* and *AIM44* gene products are known to be cell cycle regulators in both species. Despite >100 million years of divergence, both yeast species still found similar genetic and phenotypic solutions, indicating the importance of shared ancestry and conserved mechanisms of cellular reproduction in the adaptive responses. Based on our findings, we anticipate that the over 1000 known budding yeast species^25^ would likely evolve convergently phenotypically and genetically to settling selection, leading to snowflake multicellularity and loss of function mutations in cell separation. Furthermore, the loss of function mutations is expected to occur in a small number of loci, especially in *ACE2* and possibly *AIM44*.

Other studies have also demonstrated that convergence can be affected by common ancestry and similar environmental pressure^9,22,26^. The extent of convergence and divergence in evolutionary outcomes is generally less easy to assess. Evolutionary outcomes involving divergence are thought to involve chance and contingent effects of accumulated differences. The divergence in unicell co-existence with multicellular snowflakes in the two yeast systems appears contingent upon differences in floccing ability^15^. *K. lactis* snowflakes are generally smaller than *S. cerevisiae* snowflakes but form bigger settling units through floccing^15^. Floccing was key for unicell persistence as it provided an opportunity for unicells to co-settle with snowflakes and survive settling selection^15,27^. Previous experimental and comparative studies have shown that as evolving lineages accumulate more differences, the power of natural selection driving convergence is reduced and the contingent effects of history are amplified^7,8,28–31^.

### Tempo and diversity of multicellularity in *K. lactis*

The emergence of a large effect beneficial mutation in a clonally reproducing population often results in the new phenotype sweeping through the population^32,33^. In *K. lactis*, however, we observed multi-uni coexistence due to frequency-dependent selection^15^. In frequency-dependent maintenance of variation, the newly emergent snowflakes invaded rapidly and reached to a plateau of about 76% frequency when unicellular phenotypes became rare (Fig. 3). Our genomic analyses show that additional new snowflakes emerge, only sometimes replacing the previous snowflakes. While the genes associated with multicellularity in *K. lactis* had low divergence, the dynamics of newly emerging *ACE2* mutants within each lineage were quite diverse. In at least one case, excluded genotypes were early arising snowflakes outcompeted by larger-sized snowflakes with better settling ability. The repeated emergence of *ACE2* mutants indicates that loss of function mutants at this locus generally confers a greater selective benefit than the other possible mutational pathways. Exclusion could potentially have been due to clonal interference. However, previous studies have shown that the “clones” usually have a beneficial mutation in another gene^33–37^, which is not the case here. It is likely that more than one mutant arose in the mono-snowflake populations such as Y2, Y3 and Y6. We hypothesize that new mutants were not competitive enough and quickly excluded by the first snowflake within 10 days. Thus they were not detected.

Another common evolutionary outcome involving multiple mutations is refinement through new mutations. For example, the citT genotype in the LTEE greatly improved its citrate utilization efficiency with an additional mutant dctA allele^38^. We did not observe further refinement of *ACE2* or *AIM44* and all snowflakes were confirmed to be single mutants. One caveat is that the population genomic sequencing data cannot exclude the possibility of double or triple mutations occurring in one snowflake genotype. However, we have confirmed all isogenic genomes have just one mutation in *ACE2* or *AIM44*. Additionally, *ACE2*/*AIM44* mutant frequencies never reached above 100%. These results indicate that the detected *ACE2* mutants were probably separate genotypes instead of being double or triple mutants. One explanation for the lack of refinement could be that *ACE2* mutations involve a loss of function^23^. In contrast, a gain of function may allow for more subsequent adjustments to the expression level of the gene^37–39^. While constrained, phenotypic refinement in loss of function mutants may occur through changes to other genes. Isogenic genome sequencing revealed two multicellular strains from two lineages had mutations involving a second gene, *CIP1*, suggesting that some snowflake mutants may have undergone the refinement, which we are exploring.

The basis for coexistence of multiple *ACE2* variants in single populations is still uncertain, and likely depends on the interaction of later appearing snowflakes with preexisting genotypes^40–42^, potentially with life history differences in snowflake reproduction^43^. Even though floccing behavior could, in principle, have played a role, it was indiscriminate among genotypes^15,27^, and larger cluster size played a preeminent role in promoting settling and survival.

### The effect of chance likely depends on ploidy

*S. cerevisiae* and *K. lactis* are genetically very different. *S. cerevisiae* has a 24 Mb diploid genome consisting of 16 pairs of nuclear chromosomes which contains two copies of about 6300 genes, whereas *K. lactis* only has 6 nuclear chromosomes with a smaller 10.6 Mb haploid genome containing about 4800 genes^17,44,45^. Given these large genetic differences, the degree of genetic convergence between *S. cerevisiae* and *K. lactis* in snowflake multicellularity is striking. These differences are also likely responsible for greater parallel genetic evolution in *K. lactis* than *S. cerevisiae*. As genetic makeup alters future adaptive pathways, evolutionary consequences are largely related to the genetic backgrounds of two yeast species^46–48^. The dissimilar gene pool size and gene linkages restrict potential pathways for each species differently^47^ and in the effects of chance in the appearance and fixation of mutations^32,34^.

Ploidy fundamentally changes how random mutations affects phenotypes^17,18,23,49^. In haploid *K. lactis*, population genomic data showed that mutations in the *ACE2* gene occurred early, within 10 days. Consistent with other systems, the rapid emergence of snowflakes was likely promoted by the organism’s haploid genome which does not allow for the masking of mutations^50^. Hence, particularly beneficial alleles can rapidly increase in frequency. In diploid *S. cerevisiae*, the evolution of snowflake multicellularity requires more time than *K. lactis*^15,16,51^. Not only does snowflake multicellularity require more time, *S. cerevisiae* genotypes heterozygous for the *ACE2* multicellular alleles are smaller than the unicellular ancestors and correspondingly settle more slowly than their ancestor^51^. When both species undergo settling selection simultaneously, *K. lactis* evolves multicellular snowflakes while *S. cerevisiae* does not, frequently leading to the exclusion of *S. cerevisiae*^52^. Moreover, masking increases the opportunity for alternative beneficial but less advantageous alleles to arise, increasing their opportunities for chance fixation. This difference in masking is likely to lead to greater exploration of different beneficial alleles in *S. cerevisiae*, and less parallelism among replicate populations than *K. lactis*. It is worth noting that the adaptation in our case was through loss-of-function mutations. Effects of masking is strong on recessive traits due to loss-of-function mutations. Adaptation through gain-of-function mutations, often shown as dominant traits, might show a different pattern without masking effects.

Multicellularity has evolved numerous times and dramatically altered the diversity of life. Many taxa have evolved multicellular forms, five having especially complex multicellular structures^53–55^. Even within the same phylogenetic clade, the subsequent evolutionary trajectories differ with some groups remaining unicellular, some becoming multicellular, and some reverting back to being unicellular from a multicellular state^54,56^. Disentangling the evolutionary basis for the diverse forms of multicellularity is challenging, because evolutionary responses are shaped by both the selective environment and underlying genetic mechanisms^23,57–59^. Teasing apart their contributions in the evolutionary process is challenging, especially because evolutionary chance and contingency are difficult to assess for many systems. Random mutations and genetic drift could be the forces generating diversity within a clade^60^. In particular, it is almost impossible to determine the causes of variation in the evolutionary responses; whether they be due to divergent history or chance events. In two yeast species, *K. lactis* and *S. cerevisiae*, settling selection rapidly leads to the convergent evolution of snowflake multicellular phenotypes and convergent genetic changes. The convergence is likely because of shared mechanisms of cellular reproduction, even though the species differ in ploidy, chromosome and gene number and diverged over 100 mya^44,45^. At the same time, species differences also generated divergent responses, including genetic variation for multicellular alleles. The different evolutionary outcomes were mainly attributed to divergent history, which was reflected in the genetic background and phylogenies of the two species^1,47,61,62^. Our work disentangles contributions to evolutionary convergence and illustrates the potential for simultaneously divergent responses to selection.

## Methods

### Settling selection experiment

Ten parallel isogenic *K. lactis* populations (labeled as Y1, Y2 … Y10) were established from a single ancestral genotype. Details of the selection are described in the work reported by Driscoll and Travisano^15^. Daily selection was conducted for 60 days. Frozen stock of each population was created every ten transfers, with 700ul culture sample placed in 15% glycerol and stored at -80 °C.

### Characterization of phenotypes

Populations were revived from the Day 60 isogenic glycerol stocks to further characterize the phenotypes. The Day 60 populations were streaked on YPD agar plates^63^ to pick individual colonies.

Colony morphologies from agar plate growth were used to distinguish unicellular and multicellular forms, which was similar to what was observed in the previously evolved multicellular yeast *S. cerevisiae*^16^. Additionally, the 24 h life cycle was monitored for these populations. Multicellular phenotypes were identified in the populations based on the colony morphology and microscopic structures (see results). Each characterized colony was later preserved as isogenic glycerol stocks for future use.

### Naming schemes of snowflakes

For each of the ten populations, we first picked isogenic glycerol stocks for each representative phenotype present. These isolated strains were named as ‘sn’ as snowflakes and indexed. For example, two snowflake strains from the Y8 population were isolated and labeled as “Y8sn-1” and “Y8sn-2”. Strains that were confirmed by isogenic genome sequencing are shown in Table 1 as bolded.

Additional snowflake colonies from certain populations were picked for further ecological tests. Specifically, Y1sn-2 was picked from Y1 Day60 population and Y7sn-2 was from Y7 Day10 population frozen stocks.

Some putative snowflake phenotypes were detected in the population sequencing data but were not physically isolated for examination. These were simply named with their population name, possible phenotype and number following the index, such as ‘Y5sn-2’, which represented one snowflake genotype detected in the Y5 population genome data (see Table 1).

### Genomic sequencing of representative phenotypes and mutation detection

To identify the genotypes of each strain, we sequenced the genomes of representative strains in each Day60 population. The glycerol stock of every representative strain was revived on YPD agar plates. A single colony was picked to grow overnight in liquid YPD culture. Cells were then harvested for DNA extraction using the QIAGEN DNeasy Blood & Tissue Kit.

Strains were sequenced on the Illumina Nextseq2000 platform. Reads were assembled by BWA and mapped to the reference genomes of the ancestor strain Y1140 with default parameters^64^. Mutations were predicted using Freebayes^65^. SNPs were filtered for quality (QUAL > = 50) and only alleles with >90% frequency were considered valid.

Mutations of interest were identified from genomic sequencing results and re-confirmed with PCR amplification. Primers used for amplifying target sequences shown in Table 2.

**Table 2:** Primers for PCR amplification of *ACE2* and *AIM44* genes

Target gene	Primer	Primer sequence
*ACE2*	ace2F	TTCGCTAGATCCAGGAATC
	ace2R	GAATCCATACGATGTCGTAGA
*AIM44*	aim44F	AGGAATGAATGAAGAACTTG
	aim44R	AGGGAGTTATGTCCAGGA

### Population genomic sequencing

To reconstruct the evolutionary trajectories of each population, 20ul aliquots were revived from frozen stocks by growing them in liquid YPD tubes overnight. Next, the population was harvested for DNA extraction using previously described methods and population genomic sequencing was then conducted. Variants at sites with <34 sequencing depth were removed from analysis. Minor alleles with <10% frequency were removed except *ACE2* and *AIM44* alleles.

### Isogenic strain construction

The URA3 gene encodes for an enzyme that catalyzes one reaction in the synthesis of pyrimidine ribonucleotides^66^. Cells that have URA3 gene knocked out cannot grow unless uracil or uridine is added to the 5’-FOA agar plate. For example, the URA3 gene was knocked out in the Y1sn-1 background to create a marker for future tests^66^, named as ‘Y1sn-1-*URA3Δ*’, similarly for ‘Y7sn-1-*URA3Δ*’. Additional strains were constructed with knockouts of certain genes such as *ACE2* and *AIM44*, as these genes were predicted to relate to snowflakes (see results). We knocked out the *ACE2* gene by replacing it with a functional URA3 gene to create an *ACE2* knockout in the ancestral Y1140 background, named as ‘Y1140-*ACE2*::*URA3*’. Successfully engineered strains were selected using uracil^-^ YNB agar plates (with amino acids). We also created a mutant ‘Y8sn-1-*ACE2*’ by replacing the ancestor’s *ACE2* with Y8sn-1’s *ACE2 *in the ancestral background.

To be specific*, URA3* gene was knocked out from the Y1140 strain to create a *URA3* knockout strain. The PCR fragment was created using nested PCR. Upstream and downstream of *URA3* were amplified. We designed the primers to create these two PCR fragments with overlapping regions, which were used as templates for nested PCR. PCR products were run on the electrophoresis agarose gel for verification and then purified. The purified DNA was later transformed into yeast cells using electroporation. Successful knockout colonies were selected on 5’-FOA YNB agar plates with amino acid and uracil. These colonies were later raised up in YPD broth media for storage and future use.

Further edits were all based on the Y1140-*URA3Δ* strain. The DNA fragment containing *URA3* gene with the *ACE2* flanking regions was amplified using PCR. Similar protocols were taken to transform this DNA fragment into the Y1140-*URA3Δ* strain, creating Y1140-ACE2::*URA3*. Successful recombinant colonies were picked on the YNB agar plates with amino acid but without uracil. Primers are listed in Table 3.

**Table 3:** Primers used for creating engineered strains

primer	primer sequence	purpose
5′up_ura3	CAACTCCCGCCAGCGAATTGGCTACTAATGGCGGT	For upstream of *URA3*
3′up_dw	CAGTACGCCAGCGTCAATACACTCCCGTCAATTAGTTGCACCTTATACAGGAAAC	
5′up_dw	TCCCGTCAATTAGTTGCACCTTATACAGGAAACTTAATAGAACAAATCACATATTTAATC	For downstream of *URA3*
3′dw_ura	TGGCGACTTGATTTATTATAACAACGACAATAGG	
ura3ko_F	CTTATCACGACTATAAAGCCA	Nested PCR to create the *URA3* knockout DNA fragment
ura3ko_R	GTTCTACTTGATCAGAGGAGT	
5′ace2flk_ura3	ATGTCAGATCGGAACCGTACGCTGCAGGTCGACGGATCCCCCTGGCACGACAGGTTTCCC	To create *URA3* gene fragment with *ACE2* flanking regions. In order to construct Y1140-*ACE2::URA3*
3′ura3_ace2flk	TCCGTTGATATTAGCAGTTTATCGCCTCGACATCATCTGCCCAGATGCGAAGTTAAGTGC	

### Settling assay

Populations either had coexistence of multiple snowflake genotypes or had one genotype that competitively excluded all others. We used Y1, where two snowflake genotypes persisted, and Y7, where one snowflake genotype excluded the other, as representative populations to investigate the fitness of different snowflake genotypes within a population.

Overnight cultures of competing strains were combined with the 1:1 ratio to a final volume of 1.6 ml. We assayed the competing strains’ relative abundance before and after settling. To distinguish the two genotypes, one strain was engineered to be uracil deficient. For example, Y1 had two different snowflake genotypes Y1sn-1 and Y1sn-2. The *URA3* gene was knockout in Y1sn-1 to create Y1sn-1-*URA3*Δ. A fitness test was then conducted using Y1sn-1-*URA3*Δ and Y1sn-2 with 800ul of each strain added to a 2 ml Eppendorf tube. After vortexing, a 100ul sample was diluted and then plated on YPD agar plates to determine the relative abundance of both genotypes before settling. To test the settling ability, the remaining cells in the tube were moved to a 1.5 ml Eppendorf tube, allowed to settle for 7 min, and 100ul from the bottom of the tube was dilution plated on YPD agar plates. After 24 h growth on YPD plates, colonies were replica plated onto YNB agar plates without uracil to identify the non-knockout colonies. Colony counts were used to quantify population densities and composition.

The results were analyzed via three planned comparisons on isolate size: isolates from population Y1, isolates from population Y7, and comparing the isolates from populations Y1 and Y7. Each individual comparison was statistically significant (p < 0.005), as was the overall set of planned contrasts (F_3,8_ = 164.6, p < 0.0001).

### Calculating settling ability and population density changes

Population increase of a certain strain in a settling experiment was calculated as Ln(N_7 min/N_0 min), where N represents the population density of the target population. Settling ability was the important feature determining the fitness of a population. Selection rate constants (SRC) were calculated to measure and compare the settling ability of unicellular populations in a co-settling experiment^7^.

### Coulter Counter measurement of snowflake populations

To compare the snowflake sizes, population Y1and Y7 were picked as representatives. Two strains in Y1 population (Y1sn-1, Y1sn-2) and two strains in Y7 population (Y7sn-1, Y7sn-2) were measured by a Multisizer Coulter counter (Beckman Coulter). When a snowflake grows big enough, it will go through fragmentation to produce smaller snowflakes. Thus, the populations were mixed with snowflakes of different stages. Therefore, the distributions of diameters were considered as bi-modal^51,65,67^. The data was compared using the larger model which represented the big snowflakes (Supplementary Table 3).

### Floccing assay

Floccing assays were conducted using Y1 and Y7 snowflakes. Each imaged test population was comprised of 50% stained and 50% unstained snowflakes either from the same genotype (e.g. Y7sn-1) or two different genotypes (e.g. Y7sn-1 and Y7sn-2, or Y1sn-1 and Y1sn-2). To create the stained population for each genotype, a single colony was inoculated into 10 ml liquid YPD culture containing 500ul red food coloring (0.2um filter sterilized). The cells ingested the red food coloring and could be distinguishable through direct microscopic observation. The 24 h full growth cultures of both stained and unstained snowflakes were harvested and had similar cell densities. Equal volumes (500ul) of stained and unstained cultures were mixed, and then diluted 10-fold using YPD in preparation for microscopic imaging. For each population that contained two genotypes, two measurements were taken, with each genotype being stained once, to minimize the effect of staining on the outcome. The number of floccing clusters, both stained and unstained, were counted to calculate the probabilities of a stained cluster floccing with another stained or an unstained cluster.

### Reporting summary

Further information on research design is available in the Nature Portfolio Reporting Summary linked to this article.

### Supplementary information

Supplementary Information

Reporting summary

## Data Availability

Raw datasets and/or primary analyzed data of the main results were listed in the supplement tables. All sequencing files are deposited in NCBI SRA (BioProject ID PRJNA1078448). These sequencing datasets can also be accessed on request from the corresponding author.
