# Supplementary material for: Genomic sequencing reveals convergent adaptation during experimental evolution in two budding yeast species
Source: Commun Biol. 2024 Jul 7;7:825. doi: 10.1038/s42003-024-06485-y (PMC11227552; doi:10.1038/s42003-024-06485-y)
Supplement: Supplementary file 1 — Supplementary Information [file 42003_2024_6485_MOESM1_ESM.pdf]

supplemental table 1

| time | strain | freq | gene | pop |
|---|---|---|---|---|
| 0 | y1sn-1 | 0 | ace2 | 1 |
| 10 | y1sn-1 | 0.2857143 | ace2 | 1 |
| 20 | y1sn-1 | 0.3823529 | ace2 | 1 |
| 30 | y1sn-1 | 0.3 | ace2 | 1 |
| 40 | y1sn-1 | 0.1463415 | ace2 | 1 |
| 50 | y1sn-1 | 0.2352941 | ace2 | 1 |
| 60 | y1sn-1 | 0.1688312 | ace2 | 1 |
| 0 | y1sn-2 | 0 | ace2 | 1 |
| 10 | y1sn-2 | 0.5102041 | ace2 | 1 |
| 20 | y1sn-2 | 0.6315789 | ace2 | 1 |
| 30 | y1sn-2 | 0.5625 | ace2 | 1 |
| 40 | y1sn-2 | 0.4137931 | ace2 | 1 |
| 50 | y1sn-2 | 0.4477612 | ace2 | 1 |
| 60 | y1sn-2 | 0.5733333 | ace2 | 1 |
| 0 | y2sn-1 | 0 | ace2 | 2 |
| 10 | y2sn-1 | 0.7647059 | ace2 | 2 |
| 20 | y2sn-1 | 1 | ace2 | 2 |
| 30 | y2sn-1 | 0.9491525 | ace2 | 2 |
| 40 | y2sn-1 | 0.6896552 | ace2 | 2 |
| 50 | y2sn-1 | 0.6716418 | ace2 | 2 |
| 60 | y2sn-1 | 0.6666667 | ace2 | 2 |
| 0 | y3sn-1 | 0 | ace2 | 3 |
| 10 | y3sn-1 | 0 | ace2 | 3 |
| 20 | y3sn-1 | 0.9833333 | ace2 | 3 |
| 30 | y3sn-1 | 0.9183673 | ace2 | 3 |
| 40 | y3sn-1 | 0.5818182 | ace2 | 3 |
| 50 | y3sn-1 | 0.7761194 | ace2 | 3 |
| 60 | y3sn-1 | 0.7636364 | ace2 | 3 |
| 0 | y3sn-2 | 0 | ace2 | 3 |
| 10 | y3sn-2 | 0.804878 | ace2 | 3 |
| 20 | y3sn-2 | 0 | ace2 | 3 |
| 30 | y3sn-2 | 0 | ace2 | 3 |
| 40 | y3sn-2 | 0 | ace2 | 3 |
| 50 | y3sn-2 | 0 | ace2 | 3 |
| 60 | y3sn-2 | 0 | ace2 | 3 |
| 0 | y4sn-1 | 0 | ace2 | 4 |
| 10 | y4sn-1 | 0 | ace2 | 4 |
| 20 | y4sn-1 | 0 | ace2 | 4 |
| 30 | y4sn-1 | 0.2553191 | ace2 | 4 |
| 40 | y4sn-1 | 0.2586207 | ace2 | 4 |
| 50 | y4sn-1 | 0.3818182 | ace2 | 4 |
| 60 | y4sn-1 | 0.5773196 | ace2 | 4 |
| 0 | y4sn-2 | 0 | ace2 | 4 |
| 10 | y4sn-2 | 0 | ace2 | 4 |
| 20 | y4sn-2 | 0.7809524 | ace2 | 4 |

| | | | | |
|---|---|---|---|---|
| 30 | y4sn-2 | 0.5 | ace2 | 4 |
| 40 | y4sn-2 | 0.2767857 | ace2 | 4 |
| 50 | y4sn-2 | 0.2168675 | ace2 | 4 |
| 60 | y4sn-2 | 0.1818182 | ace2 | 4 |
| 0 | y4sn-3 | 0 | ace2 | 4 |
| 10 | y4sn-3 | 0.6727273 | ace2 | 4 |
| 20 | y4sn-3 | 0 | ace2 | 4 |
| 30 | y4sn-3 | 0 | ace2 | 4 |
| 40 | y4sn-3 | 0 | ace2 | 4 |
| 50 | y4sn-3 | 0 | ace2 | 4 |
| 60 | y4sn-3 | 0 | ace2 | 4 |
| 0 | y5sn-1 | 0 | ace2 | 5 |
| 10 | y5sn-1 | 0.796875 | ace2 | 5 |
| 20 | y5sn-1 | 0.8166667 | ace2 | 5 |
| 30 | y5sn-1 | 0.8117647 | ace2 | 5 |
| 40 | y5sn-1 | 0.6075949 | ace2 | 5 |
| 50 | y5sn-1 | 0.5689655 | ace2 | 5 |
| 60 | y5sn-1 | 0.734375 | ace2 | 5 |
| 0 | y5sn-2 | 0 | ace2 | 5 |
| 10 | y5sn-2 | 0 | ace2 | 5 |
| 20 | y5sn-2 | 0.125 | ace2 | 5 |
| 30 | y5sn-2 | 0.0655738 | ace2 | 5 |
| 40 | y5sn-2 | 0.0740741 | ace2 | 5 |
| 50 | y5sn-2 | 0.0416667 | ace2 | 5 |
| 60 | y5sn-2 | 0.0588235 | ace2 | 5 |
| 0 | y6sn-1 | 0 | ace2 | 6 |
| 10 | y6sn-1 | 0.7580645 | ace2 | 6 |
| 20 | y6sn-1 | 0.94 | ace2 | 6 |
| 30 | y6sn-1 | 0.74 | ace2 | 6 |
| 40 | y6sn-1 | 0.5636364 | ace2 | 6 |
| 50 | y6sn-1 | 0.6612903 | ace2 | 6 |
| 60 | y6sn-1 | 0.6428571 | ace2 | 6 |
| 0 | y7sn-1 | 0 | ace2 | 7 |
| 10 | y7sn-1 | 0 | ace2 | 7 |
| 20 | y7sn-1 | 0.9756098 | ace2 | 7 |
| 30 | y7sn-1 | 0.8617021 | ace2 | 7 |
| 40 | y7sn-1 | 0.6593407 | ace2 | 7 |
| 50 | y7sn-1 | 0.7567568 | ace2 | 7 |
| 60 | y7sn-1 | 0.6515152 | ace2 | 7 |
| 0 | y7sn-2 | 0 | ace2 | 7 |
| 10 | y7sn-2 | 0.691358 | ace2 | 7 |
| 20 | y7sn-2 | 0 | ace2 | 7 |
| 30 | y7sn-2 | 0 | ace2 | 7 |
| 40 | y7sn-2 | 0 | ace2 | 7 |
| 50 | y7sn-2 | 0 | ace2 | 7 |
| 60 | y7sn-2 | 0 | ace2 | 7 |
| 0 | y8sn-1 | 0 | ace2 | 8 |

| | | | | |
|---|---|---|---|---|
| 10 | y8sn-1 | 0 | ace2 | 8 |
| 20 | y8sn-1 | 0.2115385 | ace2 | 8 |
| 30 | y8sn-1 | 0.2658228 | ace2 | 8 |
| 40 | y8sn-1 | 0.3495935 | ace2 | 8 |
| 50 | y8sn-1 | 0.2786885 | ace2 | 8 |
| 60 | y8sn-1 | 0.6235294 | ace2 | 8 |
| 0 | y8sn-2 | 0 | ace2 | 8 |
| 10 | y8sn-2 | 0 | ace2 | 8 |
| 20 | y8sn-2 | 0.5666667 | ace2 | 8 |
| 30 | y8sn-2 | 0.5833333 | ace2 | 8 |
| 40 | y8sn-2 | 0.3761468 | ace2 | 8 |
| 50 | y8sn-2 | 0.1926606 | ace2 | 8 |
| 60 | y8sn-2 | 0.3 | ace2 | 8 |
| 0 | y9sn-2 | 0 | ace2 | 9 |
| 10 | y9sn-2 | 0.6818182 | ace2 | 9 |
| 20 | y9sn-2 | 0 | ace2 | 9 |
| 30 | y9sn-2 | 0 | ace2 | 9 |
| 40 | y9sn-2 | 0 | ace2 | 9 |
| 50 | y9sn-2 | 0 | ace2 | 9 |
| 60 | y9sn-2 | 0 | ace2 | 9 |
| 0 | y9sn-3 | 0 | ace2 | 9 |
| 10 | y9sn-3 | 0 | ace2 | 9 |
| 20 | y9sn-3 | 0.4090909 | ace2 | 9 |
| 30 | y9sn-3 | 0.4202899 | ace2 | 9 |
| 40 | y9sn-3 | 0.1929825 | ace2 | 9 |
| 50 | y9sn-3 | 0.2021277 | ace2 | 9 |
| 60 | y9sn-3 | 0.1318681 | ace2 | 9 |
| 0 | y9sn-1 | 0 | ace2 | 9 |
| 10 | y9sn-1 | 0 | ace2 | 9 |
| 20 | y9sn-1 | 0.4823529 | ace2 | 9 |
| 30 | y9sn-1 | 0.5064935 | ace2 | 9 |
| 40 | y9sn-1 | 0.3736264 | ace2 | 9 |
| 50 | y9sn-1 | 0.3921569 | ace2 | 9 |
| 60 | y9sn-1 | 0.137931 | ace2 | 9 |
| 0 | y9sn-4 | 0 | aim44 | 9 |
| 10 | y9sn-4 | 0 | aim44 | 9 |
| 20 | y9sn-4 | 0 | aim44 | 9 |
| 30 | y9sn-4 | 0 | aim44 | 9 |
| 40 | y9sn-4 | 0.0551181 | aim44 | 9 |
| 50 | y9sn-4 | 0.3052632 | aim44 | 9 |
| 60 | y9sn-4 | 0.6333333 | aim44 | 9 |
| 0 | y10sn-2 | 0 | aim44 | 10 |
| 10 | y10sn-2 | 0.0721649 | aim44 | 10 |
| 20 | y10sn-2 | 0.0082645 | aim44 | 10 |
| 30 | y10sn-2 | 0.030303 | aim44 | 10 |
| 40 | y10sn-2 | 0.0077519 | aim44 | 10 |
| 50 | y10sn-2 | 0.0322581 | aim44 | 10 |

| | | | | |
|---|---|---|---|---|
| 60 | y10sn-2 | 0.0983607 | aim44 | 10 |
| 0 | y10sn-3 | 0 | ace2 | 10 |
| 10 | y10sn-3 | 0 | ace2 | 10 |
| 20 | y10sn-3 | 0.1538462 | ace2 | 10 |
| 30 | y10sn-3 | 0.0630631 | ace2 | 10 |
| 40 | y10sn-3 | 0.0825688 | ace2 | 10 |
| 50 | y10sn-3 | 0.0843373 | ace2 | 10 |
| 60 | y10sn-3 | 0.0731707 | ace2 | 10 |
| 0 | y10sn-4 | 0 | ace2 | 10 |
| 10 | y10sn-4 | 0.0149254 | ace2 | 10 |
| 20 | y10sn-4 | 0.4545455 | ace2 | 10 |
| 30 | y10sn-4 | 0.6507937 | ace2 | 10 |
| 40 | y10sn-4 | 0.557377 | ace2 | 10 |
| 50 | y10sn-4 | 0.3846154 | ace2 | 10 |
| 60 | y10sn-4 | 0.4337349 | ace2 | 10 |
| 0 | y10sn-5 | 0 | ace2 | 10 |
| 10 | y10sn-5 | 0 | ace2 | 10 |
| 20 | y10sn-5 | 0.1971831 | ace2 | 10 |
| 30 | y10sn-5 | 0.1428571 | ace2 | 10 |
| 40 | y10sn-5 | 0.2266667 | ace2 | 10 |
| 50 | y10sn-5 | 0.1451613 | ace2 | 10 |
| 60 | y10sn-5 | 0.1650485 | ace2 | 10 |

supplement table 2

| Population | Time | # of Genotype | Snowflake Frequency |
|---|---|---|---|
| 1 | 0 | 0 | 0 |
| 1 | 10 | 2 | 0.795918368 |
| 1 | 20 | 2 | 1.014 |
| 1 | 30 | 2 | 0.8625 |
| 1 | 40 | 2 | 0.560134566 |
| 1 | 50 | 2 | 0.683055312 |
| 1 | 60 | 2 | 0.742164502 |
| 2 | 0 | 0 | 0 |
| 2 | 10 | 1 | 0.764705882 |
| 2 | 20 | 1 | 1 |
| 2 | 30 | 1 | 0.949152542 |
| 2 | 40 | 1 | 0.689655172 |
| 2 | 50 | 1 | 0.671641791 |
| 2 | 60 | 1 | 0.666666667 |
| 3 | 0 | 0 | 0 |
| 3 | 10 | 0 | 0.804878049 |
| 3 | 20 | 1 | 0.983333333 |
| 3 | 30 | 1 | 0.918367347 |
| 3 | 40 | 1 | 0.581818182 |
| 3 | 50 | 1 | 0.776119403 |
| 3 | 60 | 1 | 0.763636364 |
| 4 | 0 | 0 | 0 |
| 4 | 10 | 1 | 0.672727273 |
| 4 | 20 | 2 | 0.780952381 |
| 4 | 30 | 3 | 0.755319149 |
| 4 | 40 | 3 | 0.535406404 |
| 4 | 50 | 3 | 0.598685652 |
| 4 | 60 | 3 | 0.75913777 |
| 5 | 0 | 0 | 0 |
| 5 | 10 | 1 | 0.796875 |
| 5 | 20 | 2 | 0.941666667 |
| 5 | 30 | 2 | 0.877338476 |
| 5 | 40 | 2 | 0.681669011 |
| 5 | 50 | 2 | 0.610632184 |
| 5 | 60 | 2 | 0.793198529 |
| 6 | 0 | 0 | 0 |
| 6 | 10 | 1 | 0.758064516 |
| 6 | 20 | 1 | 0.94 |
| 6 | 30 | 1 | 0.74 |
| 6 | 40 | 1 | 0.563636364 |
| 6 | 50 | 1 | 0.661290323 |
| 6 | 60 | 1 | 0.642857143 |

| | | | |
|---|---|---|---|
| 7 | 0 | 0 | 0 |
| 7 | 10 | 1 | 0.691358025 |
| 7 | 20 | 2 | 0.975609756 |
| 7 | 30 | 2 | 0.861702128 |
| 7 | 40 | 2 | 0.659340659 |
| 7 | 50 | 2 | 0.756756757 |
| 7 | 60 | 2 | 0.651515152 |
| 8 | 0 | 0 | 0 |
| 8 | 10 | 0 | 0 |
| 8 | 20 | 2 | 0.778205129 |
| 8 | 30 | 2 | 0.849156118 |
| 8 | 40 | 2 | 0.725740285 |
| 8 | 50 | 2 | 0.471349075 |
| 8 | 60 | 2 | 0.923529412 |
| 9 | 0 | 0 | 0 |
| 9 | 10 | 1 | 0.681818182 |
| 9 | 20 | 3 | 0.89144385 |
| 9 | 30 | 3 | 0.926783361 |
| 9 | 40 | 4 | 0.62172694 |
| 9 | 50 | 4 | 0.899547681 |
| 9 | 60 | 4 | 0.903132499 |
| 10 | 0 | 0 | 0 |
| 10 | 10 | 2 | 0.087090321 |
| 10 | 20 | 4 | 0.813839171 |
| 10 | 30 | 4 | 0.887016887 |
| 10 | 40 | 4 | 0.874364461 |
| 10 | 50 | 4 | 0.646372089 |
| 10 | 60 | 4 | 0.770314872 |

supplement table 3 coulter counter bimodal data

| | | | | | |
|---|---|---|---|---|---|
| y7sn-2 rep1 | D = 0.042525, p-value < 2.2e-16 | y7sn-2 rep2 | D = 0.047835, p-value < 2.2e-16 | y7sn-2 rep3 | = 0.039358, p-value < 2.2e-16 |
| summary of normalmixEM object: | | summary of normalmixEM object: | | summary of normalmixEM object: | alternative hypothesis: non-unimodal, i.e., at least bimodal |
| comp 1 comp 2 | | comp 1 comp 2 | | comp 1 comp 2 | |
| lambda 0.709567 0.290433 | | lambda 0.732822 0.267178 | | lambda 0.770337 0.229663 | |
| mu 3.554448 19.768664 | | mu 3.575836 20.892110 | | mu 3.586680 20.393048 | |
| sigma 0.260535 6.529857 | | sigma 0.283128 5.779751 | | sigma 0.277294 6.330059 | |
| loglik at estimate: -6070605 | | loglik at estimate: -11297.95 | | loglik at estimate: -10651.14 | |
| plot(m1.s,which=2) | | | | | |
| y7sn-1 rep1 | D = 0.055203, p-value < 2.2e-16 | y7sn-1 rep2 | D = 0.042249, p-value < 2.2e-16 | y7sn-1 rep3 | D = 0.030137, p-value < 2.2e-16 |
| summary of normalmixEM object: | | summary of normalmixEM object: | | summary of normalmixEM object: | |
| comp 1 comp 2 | | comp 1 comp 2 | | comp 1 comp 2 | |
| lambda 0.211263 0.788737 | | lambda 0.156962 0.843038 | | lambda 0.130831 0.869169 | |
| mu 4.920451 24.406765 | | mu 5.233514 24.509000 | | mu 5.335933 24.983568 | |
| sigma 1.704450 4.497272 | | sigma 1.461376 4.533078 | | sigma 1.732751 4.724201 | |
| loglik at estimate: -5528.775 | | loglik at estimate: -2525.105 | | loglik at estimate: -4564.309 | |
| y1sn-1 rep1 | D = 0.035823, p-value < 2.2e-16 | y1sn-1 rep2 | D = 0.033639, p-value < 2.2e-16 | y1sn-1 rep3 | D = 0.028153, p-value = 2.185e-06 |
| ummary of normalmixEM object: | | summary of normalmixEM object: | | ummary of normalmixEM object: | |
| comp 1 comp 2 | | comp 1 comp 2 | | comp 1 comp 2 | |
| lambda 0.132316 0.867684 | | lambda 0.133595 0.866405 | | lambda 0.114517 0.885483 | |
| mu 5.381051 25.933884 | | mu 5.891709 26.086185 | | mu 5.513257 25.910382 | |
| sigma 1.641669 3.956961 | | sigma 1.846308 3.971035 | | sigma 1.854316 4.037225 | |
| loglik at estimate: -3755.773 | | loglik at estimate: -4409.309 | | loglik at estimate: -3621.728 | |
| y1sn-2 rep1 | D = 0.028868, p-value < 2.2e-16 | y1sn-2 rep2 | D = 0.022852, p-value = 5.57e-05 | y1sn-2 rep3 | D = 0.026351, p-value < 2.2e-16 |
| summary of normalmixEM object: | | summary of normalmixEM object: | | summary of normalmixEM object: | |
| comp 1 comp 2 | | comp 1 comp 2 | | comp 1 comp 2 | |
| lambda 0.872145 0.127855 | | lambda 0.107063 0.892937 | | lambda 0.121977 0.878023 | |
| mu 24.963066 5.578172 | | mu 5.520173 24.720689 | | mu 5.818541 24.695139 | |
| sigma 4.733075 1.683679 | | sigma 1.617055 4.747649 | | sigma 1.697885 4.651585 | |
| loglik at estimate: -2751229 | | loglik at estimate: -4260.839 | | loglik at estimate: -4879.844 | |

supplement table 4 flocccing data

| red stained strain | unstained strain | data | | | | analysis | | | |
|---|---|---|---|---|---|---|---|---|---|
| y7sn-2 | y7sn-2 | | red | white | total | Chi By | Chi both | Chi | stat |
| | | OR | 26 | 57 | 83 | 0.0068815 | 0.00870141 | 0.0155829 | 0.90065697 |
| | | AND | 34 | 33 | 67 | | | | |
| | | total | 60 | 90 | 150 | | | | |
| y7sn-1 | y7sn-2 | | red | white | total | 2.2488966 | 2.60494156 | 4.85383811 | 0.02758469 |
| | | OR | 28 | 42 | 70 | | | | |
| | | AND | 42 | 44 | 86 | | | | |
| | | total | 70 | 86 | 156 | | | | |
| y7sn-1 | y7sn-1 | | red | white | total | 0.6183246 | 0.9417049 | 1.56002948 | 0.2116609 |
| | | OR | 22 | 93 | 115 | | | | |
| | | AND | 43 | 47 | 90 | | | | |
| | | total | 65 | 140 | 205 | | | | |
| y7sn-2 | y7sn-1 | | red | white | total | 0.0406582 | 0.05527241 | 0.09593056 | 0.75676888 |
| | | OR | 51 | 189 | 240 | | | | |
| | | AND | 85 | 97 | 182 | | | | |
| | | total | 136 | 286 | 422 | | | | |
| y1sn-1 | y1sn-1 | | red | white | total | 1.1116579 | 1.55025973 | 2.6619176 | 0.10277676 |
| | | OR | 46 | 75 | 121 | | | | |
| | | AND | 62 | 62 | 124 | | | | |
| | | total | 108 | 137 | 245 | | | | |
| y1sn-1 | y1sn-2 | | red | white | total | 0.0073571 | 0.0109919 | 0.01834902 | 0.89224933 |
| | | OR | 21 | 48 | 69 | | | | |
| | | AND | 22 | 23 | 45 | | | | |
| | | total | 43 | 71 | 114 | | | | |
| y1sn-2 | y1sn-1 | | red | white | total | 0.4131977 | 0.60790962 | 1.02110735 | 0.31225649 |
| | | OR | 13 | 45 | 58 | | | | |
| | | AND | 27 | 21 | 48 | | | | |
| | | total | 40 | 66 | 106 | | | | |
| | | | red | white | total | | | | |
| y1sn-2 | y1sn-2 | | red | white | total | 0.4113811 | 0.50568512 | 0.91706623 | 0.33824646 |
| | | OR | 24 | 71 | 95 | | | | |
| | | AND | 45 | 44 | 89 | | | | |
| | | total | 69 | 115 | 184 | | | | |
