# Supplementary material for: Genomic sequencing reveals convergent adaptation during experimental evolution in two budding yeast species
Source: Commun Biol. 2024 Jul 7;7:825. doi: 10.1038/s42003-024-06485-y (PMC11227552; doi:10.1038/s42003-024-06485-y)
Supplement: Supplementary file 2 — Reporting summary [file 42003_2024_6485_MOESM2_ESM.pdf]

nature portfolio

Corresponding author(s): COMMSBIO-24-0931B Pu Wang

Last updated by author(s): Jun 19, 2024

Reporting Summary

Nature Portfolio wishes to improve the reproducibility of the work that we publish. This form provides structure for consistency and transparency in reporting. For further information on Nature Portfolio policies, see our Editorial Policies and the Editorial Policy Checklist.

Statistics

For all statistical analyses, confirm that the following items are present in the figure legend, table legend, main text, or Methods section.

n/a	Confirmed	
☐	☒	The exact sample size (*n*) for each experimental group/condition, given as a discrete number and unit of measurement
☐	☒	A statement on whether measurements were taken from distinct samples or whether the same sample was measured repeatedly
☐	☒	The statistical test(s) used AND whether they are one- or two-sided<br>*Only common tests should be described solely by name; describe more complex techniques in the Methods section.*
☐	☒	A description of all covariates tested
☐	☒	A description of any assumptions or corrections, such as tests of normality and adjustment for multiple comparisons
☐	☒	A full description of the statistical parameters including central tendency (e.g. means) or other basic estimates (e.g. regression coefficient) AND variation (e.g. standard deviation) or associated estimates of uncertainty (e.g. confidence intervals)
☐	☒	For null hypothesis testing, the test statistic (e.g. F, t, r) with confidence intervals, effect sizes, degrees of freedom and P value noted<br>*Give P values as exact values whenever suitable.*
☒	☐	For Bayesian analysis, information on the choice of priors and Markov chain Monte Carlo settings
☒	☐	For hierarchical and complex designs, identification of the appropriate level for tests and full reporting of outcomes
☐	☒	Estimates of effect sizes (e.g. Cohen's d, Pearson's r), indicating how they were calculated

Our web collection on statistics for biologists contains articles on many of the points above.

Software and code

Policy information about availability of computer code

Data collection	no software used
Data analysis	ImageJ, JMP, r studio

For manuscripts utilizing custom algorithms or software that are central to the research but not yet described in published literature, software must be made available to editors and reviewers. We strongly encourage code deposition in a community repository (e.g. GitHub). See the Nature Portfolio guidelines for submitting code & software for further information.

Data

Policy information about availability of data

All manuscripts must include a data availability statement. This statement should provide the following information, where applicable:

- Accession codes, unique identifiers, or web links for publicly available datasets
- A description of any restrictions on data availability
- For clinical datasets or third party data, please ensure that the statement adheres to our policy

Raw datasets and/or primary analyzed data of the main results were listed in the supplement tables. All sequencing files are deposited in NCBI SRA (BioProject ID PRJNA1078448). These sequencing datasets can also be accessed on request from the corresponding author.

Research involving human participants, their data, or biological material

Policy information about studies with human participants or human data. See also policy information about sex, gender (identity/presentation), and sexual orientation and race, ethnicity and racism.

Reporting on sex and gender	NA
Reporting on race, ethnicity, or other socially relevant groupings	NA
Population characteristics	NA
Recruitment	NA
Ethics oversight	NA

Note that full information on the approval of the study protocol must also be provided in the manuscript.

Field-specific reporting

Please select the one below that is the best fit for your research. If you are not sure, read the appropriate sections before making your selection.

☐ Life sciences ☐ Behavioural & social sciences ☒ Ecological, evolutionary & environmental sciences

For a reference copy of the document with all sections, see nature.com/documents/nr-reporting-summary-flat.pdf

Ecological, evolutionary & environmental sciences study design

All studies must disclose on these points even when the disclosure is negative.

Study description	experimental evolution of the dairy yeast populations. The evolved 10 yeast populations were subjected to genome sequencing. The result was compared with a previously published work to reflection some evolutionary insights.
Research sample	10 yeast populations, each population has 6 frozen stocks. I also collected single isolates (1 or 2) for each population, 14 total. 1 ancestral strain.
Sampling strategy	all samples were sequenced
Data collection	PW collected the data. Samples were subjected to genome sequencing.
Timing and spatial scale	each yeast population has 6 frozen stocks for 6 time points (every 10 days, for 60 days of treatment)<br>the experiment is conducted in the lab
Data exclusions	no data point was excluded
Reproducibility	for wet lab experiment test, such as settling test and coulter-counter measurement, three replications were conducted.
Randomization	all samples were collected
Blinding	all samples were collected

Did the study involve field work? ☐ Yes ☒ No

Reporting for specific materials, systems and methods

We require information from authors about some types of materials, experimental systems and methods used in many studies. Here, indicate whether each material, system or method listed is relevant to your study. If you are not sure if a list item applies to your research, read the appropriate section before selecting a response.

Materials & experimental systems

n/a	Involved in the study	
☒	☐	Antibodies
☐	☒	Eukaryotic cell lines
☒	☐	Palaeontology and archaeology
☒	☐	Animals and other organisms
☒	☐	Clinical data
☒	☐	Dual use research of concern
☒	☐	Plants

Methods

n/a	Involved in the study	
☒	☐	ChIP-seq
☒	☐	Flow cytometry
☒	☐	MRI-based neuroimaging

Eukaryotic cell lines

Policy information about cell lines and Sex and Gender in Research

Cell line source(s)	yeast strain Y1140 was acquired from Driscoll and Travisano, 2017
Authentication	NA, it is a lab yeast strain, non pathogenic
Mycoplasma contamination	NA, it is a lab yeast strain, non pathogenic
Commonly misidentified lines (See ICLAC register)	NA, it is a lab yeast strain, non pathogenic

Plants

Seed stocks	no plants used in the project
Novel plant genotypes	no plants used in the project
Authentication	no plants used in the project
